# Dual-Band Branch-Line Coupler Based on Crossed Lines for Arbitrary Power-Split Ratios

**DOI:** 10.3390/s22155527

**Published:** 2022-07-25

**Authors:** Hyungjun Chang, Taejun Lim, Kristian Chavdarov Dimitrov, Yongshik Lee

**Affiliations:** 1 RFcore, Seongnnam 13510, Korea; hyungjunchang@yonsei.ac.kr; 2 Electronics Mobile Division, Samsung, Suwon 16877, Korea; taejunim@yonsei.ac.kr; 3 Department of Electrical and Electronic Engineering, Yonsei University, Seoul 03722, Korea; kristian.dimitrov.eng@gmail.com

**Keywords:** branch-line coupler, dual-band, crossed lines, open-ended stub, arbitrary power-split

## Abstract

Dual-band branch-line couplers with arbitrary power-split ratios are presented. The use of crossed lines at the center of the dual-band coupler enables it to independently provide different power-split ratios to the two bands. Additionally, open stubs are utilized to enhance the stopband responses. The complete design procedure with example design curves is provided. For experimental verification, three dual-band couplers with power-split ratio combinations of +3 dB ($S_{21}:S_{31}=2:1$) and −3 dB ($S_{21}:S_{31}=1:2$), −3 dB and +3 dB, and 0 dB ($S_{21}:S_{31}=1:1$) and +13 dB ($S_{21}:S_{31}=20:1$) at 1 GHz and 2.5 GHz were designed and fabricated. The measured results are in excellent agreement with the ideal and full-wave simulated results. The measured difference of −13.3 dB between the power-split ratios of the two bands is the largest reported for a dual-band branch-line coupler.

## 1. Introduction

Dual-band branch-line couplers with arbitrary power-split ratios [1,2,3,4,5,6,7,8,9,10] are being studied on an ongoing basis, because they play an important role in dual-band applications such as antenna array systems [11,12] and the Doherty power amplifier [13,14]. For example, a coupler with a large power-split ratio can be utilized in a butler-matrix-based very large antenna array to reduce the side lobe level [15]. Various techniques have been used to develop couplers with the required capabilities. For example, tightly coupled lines were utilized to develop a dual-band coupler with an arbitrary power-split ratio, with a frequency ratio as large as 11.7 [4]. Apart from this, three parallel lines [5], bridged-T coils [6], and a stepped-impedance section with open stubs [7] have been successfully employed to obtain dual-band couplers. However, these couplers are problematic in that they either require the two bands to have the same power-split ratios [5], or the difference between the ratios in the two bands is insufficiently small [6]. A dual-band coupler with a difference in power-split ratios as large as 10.2 dB ($S_{21}:S_{31}=10.5:1$) has been presented [8]. However, integrated complimentary split-ring resonators may require time-consuming full-wave simulations and have limited frequency scalability.

In this paper, we present dual-band branch-line couplers with arbitrary power-split ratios based on crossed lines, which makes it possible to obtain a very large difference between the arbitrary power-split ratios in the two bands. Further, the open stubs are utilized to provide design freedom that not only widens the range of practical power and/or frequency split ratios, but also improves the bandwidths, and enhances the stopband response. The proposed method was verified by conducting experiments with three dual-band couplers. The experimental results are in excellent agreement with the simulated results. The power-split ratios of the two bands were measured to differ by as much as 13.3 dB ($S_{21}:S_{31}=21.4:1$), which is the largest difference reported thus far.

## 2. Design Procedure

**Figure 1:**
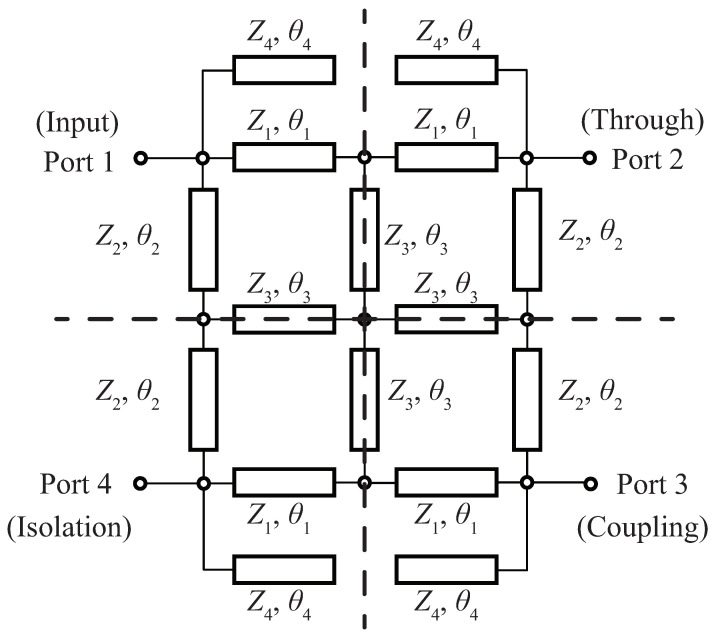
Schematic of proposed dual-band coupler with crossed lines and open-ended stubs. Dotted lines are planes of symmetry.

A schematic of the proposed dual-band branch-line coupler is shown in Figure 1. The four lines of the conventional branch-line coupler with characteristic impedances ($Z_{1}$, $Z_{2}$) and electrical lengths ($\theta_{1}$, $\theta_{2}$) are connected with crossed lines ($Z_{3}$, $\theta_{3}$), and open-ended stubs ($Z_{4}$, $\theta_{4}$) are attached to each port. In this type of circuit, dual-band operation is achieved by setting all electrical lengths to 180${}^{\circ }$/$\left(n+1\right)$ at $f_{1}$, where $n=f_{2}/f_{1}$ is the ratio of the center frequencies [5]. However, in this work, to accomplish arbitrary power-split ratios in the two bands, this constraint is abandoned and all electrical lengths remain as variables.

**Figure 2:**
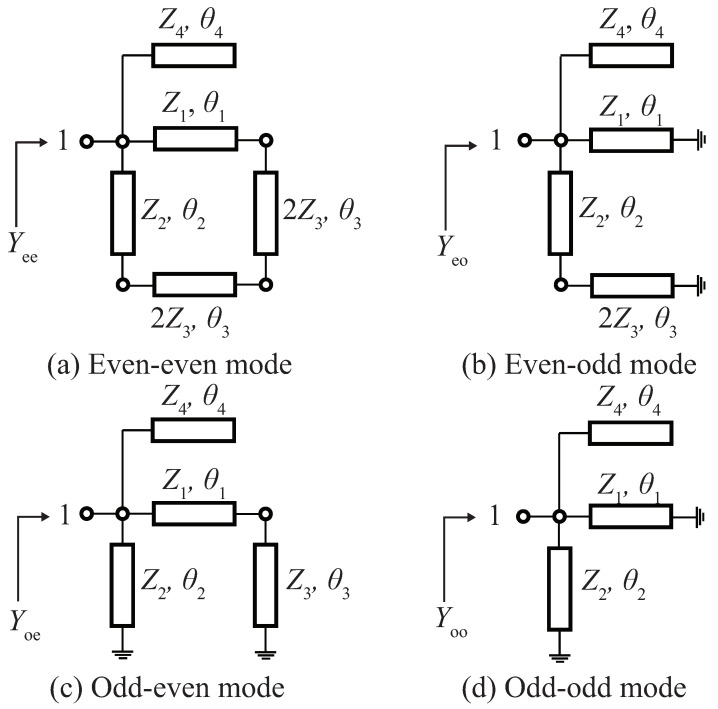
Equivalent circuits of the proposed coupler.

By applying consecutive even- and odd-mode analysis between Port 1 and Port 4, and then Port 1 and Port 2 (or Port 4 and Port 3), the two planes of symmetry in Figure 1 becomes the electric and magnetic walls, resulting in the four equivalent circuits in Figure 2. The input admittances are obtained, and $Y_{i}$ (*i* = 1, 2, 3, 4) are the characteristic admittances.

(1) $$Y_{ee}=A-\frac{C^{2}}{B}+jY_{4}\tan\theta_{4},$$

(2) $$Y_{eo}=j\left(\frac{Y_{2}\left(2Y_{2}\tan\theta_{2}-Y_{3}\cot\theta_{3}\right)}{2Y_{2}+Y_{3}\cot\theta_{3}\tan\theta_{2}}-Y_{1}\cot\theta_{1}+Y_{4}\tan\theta_{4}\right),$$

(3) $$Y_{oe}=j\left(\frac{Y_{1}\left(2Y_{1}\tan\theta_{1}-Y_{3}\cot\theta_{3}\right)}{2Y_{1}+Y_{3}\cot\theta_{3}\tan\theta_{1}}-Y_{2}\cot\theta_{2}+Y_{4}\tan\theta_{4}\right),$$

(4) $$Y_{oo}=-j\left(Y_{1}\cot\theta_{1}+Y_{2}\cot\theta_{2}-Y_{4}\tan\theta_{4}\right),$$

where

(5) $$A=j(\frac{Y_{1}\left(2Y_{1}\tan\theta_{1}-Y_{3}\cot\theta_{3}\right)}{2Y_{1}+Y_{3}\tan\theta_{1}\cot\theta_{3}}+\frac{Y_{2}\left(2Y_{2}\tan\theta_{2}-Y_{3}\cot\theta_{3}\right)}{2Y_{2}+Y_{3}\tan\theta_{2}\cot\theta_{3}}),$$

(6) $$B=j(\frac{Y_{3}\left(Y_{3}\tan\theta_{3}-2Y_{1}\cot\theta_{1}\right)}{2Y_{3}+4Y_{1}\tan\theta_{3}\cot\theta_{1}}+\frac{Y_{3}\left(Y_{3}\tan\theta_{3}-2Y_{2}\cot\theta_{2}\right)}{2Y_{3}+4Y_{2}\tan\theta_{3}\cot\theta_{2}}),$$

(7) $$C=j(\frac{Y_{1}Y_{3}}{2Y_{1}\cos\theta_{1}\sin\theta_{3}+Y_{3}\cos\theta_{3}\sin\theta_{1}}-\frac{Y_{2}Y_{3}}{2Y_{2}\cos\theta_{2}\sin\theta_{3}+Y_{3}\cos\theta_{3}\sin\theta_{2}}).$$

Once the *Y* parameters are converted to the *S* parameters, the ideal conditions for a dual-band coupler, that is, $S_{11}$ = 0 and $S_{41}$ = 0, and $S_{21}\;=\;j\sqrt{k_{1}}S_{31}$ at *f* = $f_{1}$ and $S_{21}\;=\;-j\sqrt{k_{2}}S_{31}$ at *f* = $f_{2}$, can be applied. If necessary, different phase conditions can be applied. The results are the following three equations that must be satisfied at the two frequencies:

(8) $$\left\{\begin{array}{c} Y_{ee}Y_{eo}=Y_{0}^{2},\;Y_{oe}Y_{oo}=Y_{0}^{2},\\ Y_{ee}Y_{oe}=Y_{0}^{2}\pm j\sqrt{k_{m}}Y_{0}^{2}\left(Y_{ee}-Y_{oe}\right). \end{array}\right.$$

where $Y_{0}$ is the system admittance, and *m* = 1, 2. Alternatively, the last condition in (8) can be replaced by $Y_{eo}Y_{oo}={Y_{o}}^{2}\pm j\sqrt{k_{m}}Y_{o}\left(Y_{eo}-Y_{oo}\right)$, which will yield the same results. For the last condition in (8), the sign is positive when *m* = 1, or at $f=f_{1}$ and negative when *m* = 2, or at $f=f_{2}$, which is due to the phase relationship between the two outputs in the two bands. The characteristic impedance $Z_{4}$ of the open stubs is set as a free variable that provides design freedom. However, its electrical length is set to $\theta_{4}$ = 180${}^{\circ }$/$\left(n+1\right)$ at $f_{1}$ to improve the stopband response with little effect on the passband performance. With predetermined power-split ratios ($k_{1}$, $k_{2}$), and the open stub parameters ($Z_{4}$, $\theta_{4}$), (8) is solved to obtain the complete design parameters.

**Figure 3:**
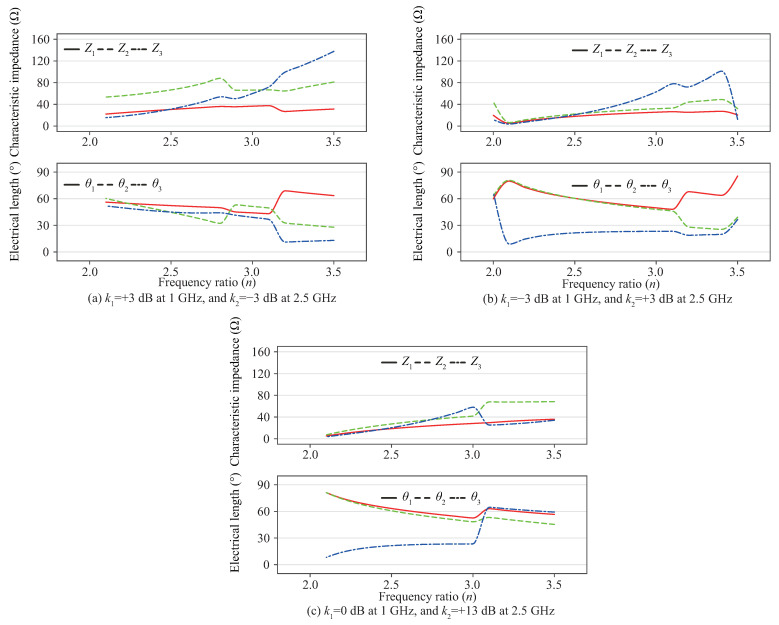
Design curves with respect to center frequency ratio *n* when $Z_{4}$ = 50 Ω and $\theta_{4}$ = 180${}^{\circ }$/$\left(n+1\right)$.

Figure 3 shows examples of the design curves for power-split ratio combinations of $k_{1}$ = +3 dB and $k_{2}$ = −3 dB, $k_{1}$ = −3 dB and $k_{2}$ = +3 dB, and $k_{1}$ = 0 dB and $k_{2}$ = +13 dB when $Z_{4}$ = 50 Ω, which implies that the proposed dual-band coupler can be designed with a large difference in the coupling in the two bands for a very wide range of *n*. Although it is not shown here, $Z_{4}$ has a notable effect on the impedance and electrical lengths of the other three lines. For example, for the $k_{1}$ = −3 dB and $k_{2}$ = +3 dB coupler, the characteristic impedances are $Z_{1}$ = 17.8 Ω, $Z_{2}$ = 22.2 Ω, and $Z_{3}$ = 20.8 Ω when $Z_{4}$ = 50 Ω and $\theta_{4}$ = 180${}^{\circ }$/$\left(n+1\right)$, where *n* = 2.5. When $\theta_{4}$ = 180${}^{\circ }$/$\left(n+1\right)$ where *n* = 2.5, the impedances increase to more practical values of 25.1 Ω, 31.3 Ω, and 52.7 Ω, when $Z_{4}$ increases from 50 Ω to 155 Ω. Additionally, special techniques [16,17,18,19] can be adopted to expand the range of realizable impedance levels of microstrip lines. Similarly, $Z_{4}$ can be varied when the electrical lengths are too long or too short. Thus in this work, the stubs provide not only an enhanced stopband response, but also additional design freedom. However, when the differences between the electrical lengths $\theta_{1}$, $\theta_{2}$, and $\theta_{3}$ are too large, the coupler may suffer from unwanted coupling between transmission lines or may not be implementable.

## 3. Simulation and Experimental Results

**Figure 4:**
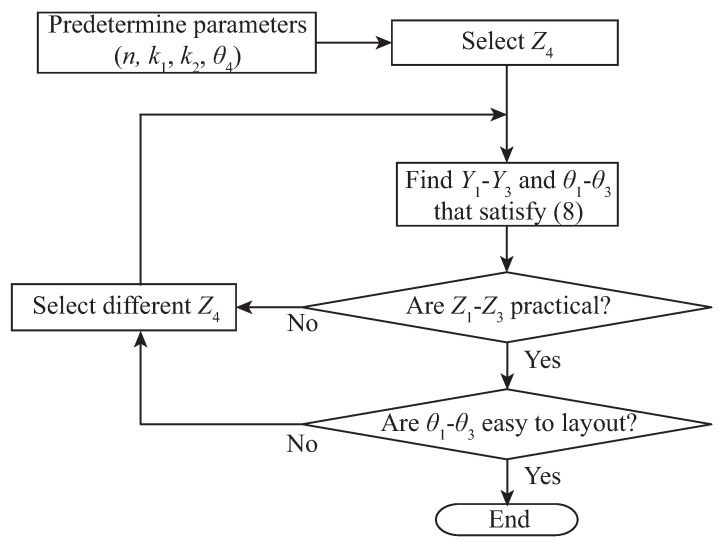
Design flow chart for proposed coupler.

Experimental verification was conducted by designing three dual-band couplers. While the proposed couplers can be designed to provide various combinations of coupling levels and the two center frequencies, the three couplers are designed to have $k_{1}$ = +3 dB and $k_{2}$ = −3 dB (Coupler A), $k_{1}$ = −3 dB and $k_{2}$ = +3 dB (Coupler B), and $k_{1}$ = 0 dB and $k_{2}$ = +13 dB (Coupler C) at 1 GHz and 2.5 GHz. Although not shown here, the proposed couplers can be designed to have a frequency ration *n* from around 2 to up to 3.5 or higher. In this work, *n* = 2.5 GHz is chosen for all three couplers, because it is between two harmonic numbers. Figure 4 show a flow chart that describes the design procedure, where all electrical lengths are evaluated at $f\;=\;f_{1}$. For example, for Coupler A, $Z_{4}$ = $1/Y_{4}$ = 160 Ω and $\theta_{4}$ = 180${}^{\circ }$/$\left(n+1\right)$ = 51.4${}^{\circ }$ were selected first, where *n* = 2.5. With predetermined power-split ratios ($k_{1}$, $k_{2}$), and the open stub parameters ($Z_{4}$, $\theta_{4}$), a total of six variables $Z_{1}$∼$Z_{3}$ and $\theta_{1}$∼$\theta_{3}$ remain to be determined. Since the three conditions of an ideal coupler in (8) must be satisfied at two frequencies, a total of six equations are solved using a genetic algorithm to obtain the six design parameters. The initially calculated impedances were $Z_{1}$ = 44.9 Ω, $Z_{2}$ = 104.3 Ω and $Z_{3}$ = 109.4 Ω, and the electrical lengths were $\theta_{1}$ = 57.4${}^{\circ }$, $\theta_{2}$ = 33.6${}^{\circ }$ and $\theta_{3}$ = 54.8${}^{\circ }$. Although all impedances are in a practical range, $\theta_{2}$ is too short compared to $\theta_{1}$ and $\theta_{3}$, which may make the layout problematic, Thus the entire procedure is repeated for a different $Z_{4}$. The final parameters are calculated with $Z_{4}$ = 50 Ω, which are summarized in Table 1 along with the design parameters for the other two couplers. While the stub lengths are $\theta_{4}$ = 180${}^{\circ }$/$\left(n+1\right)$ = 51.4${}^{\circ }$ for all couplers since *n* = 2.5, the stub impedances are different. For Coupler A, $Z_{4}$ = 50 Ω is chosen, which is increased to $Z_{4}$ = 155 Ω for Coupler B and $Z_{4}$ = 100 Ω for Coupler C to maintain other impedances $Z_{1}$–$Z_{3}$ within practical levels and the electrical lengths $\theta_{1}$–$\theta_{3}$ such that layout is possible.

**Figure 5:**
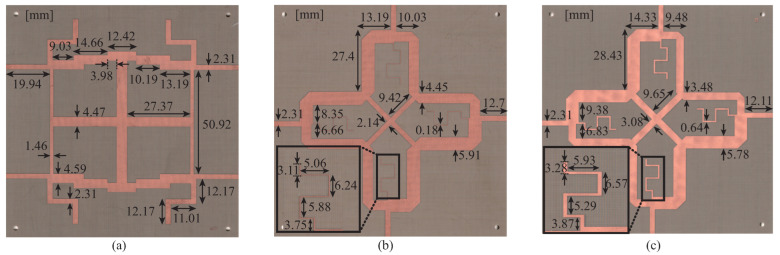
Photographic image of fabricated couplers. (**a**) Coupler A, (**b**) Coupler B, and (**c**) Coupler C.

The couplers were fabricated on a 0.76 mm thick Taconic TLY-5 substrate with $\epsilon_{r}$ = 2.2. Figure 5 shows a photographic image of the fabricated couplers with their final dimensions, which are optimized in HFSS [20].

**Figure 6:**
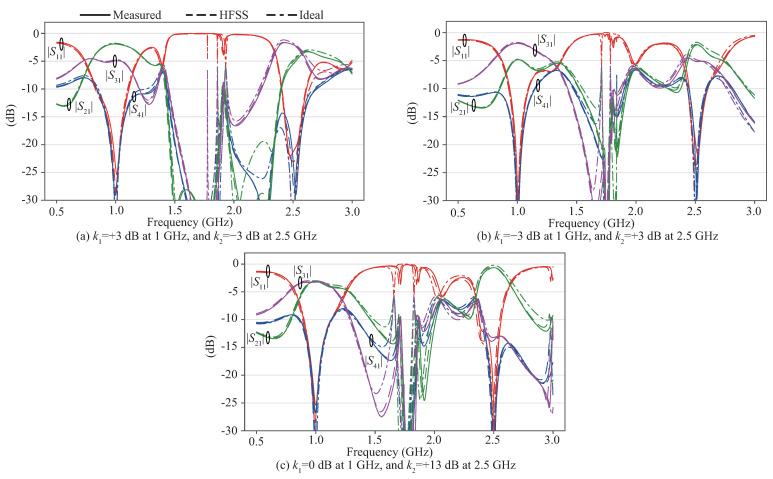
Measured, full-wave, and ideal circuit simulated results. (**a**) Coupler A, (**b**) Coupler B, and (**c**) Coupler C.

**Figure 7:**
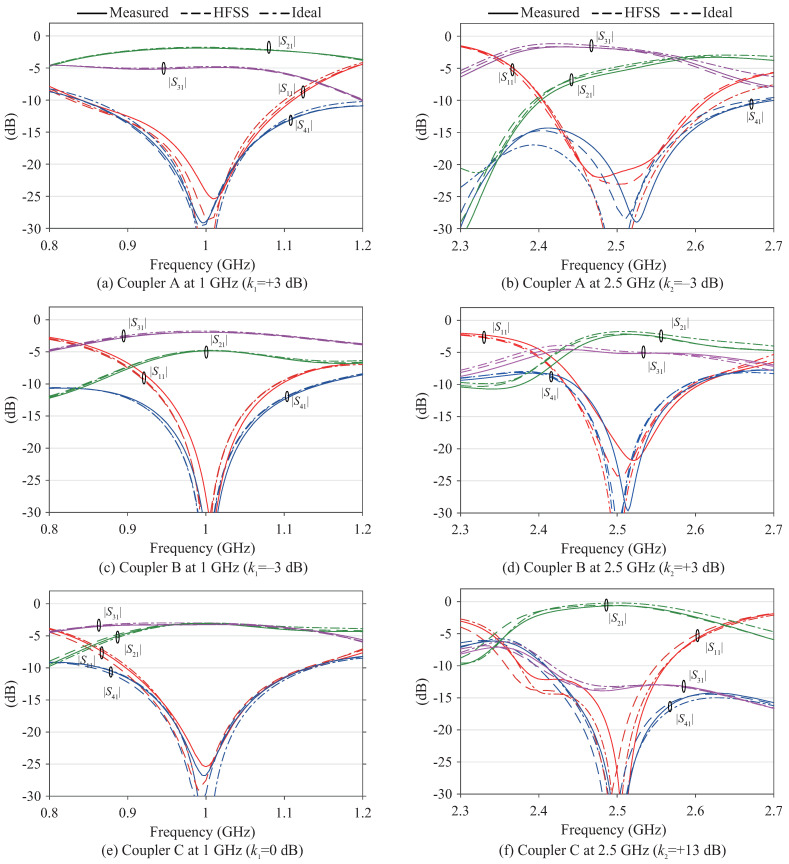
Zoom-in version of measured, full-wave, and ideal circuit simulated results.

The fabricated couplers were measured with a ZNB8 vector network analyzer from Rohde & Schwarz, which was calibrated using a ZV-Z135 calibration kit. The results are shown in Figure 6 for the entire measured band, as well as in Figure 7 that focus on the results around the two center frequencies. Comparison with the ideal and full-wave simulated results from HFSS revealed excellent agreement between experiment and theory, with successful enhancement of the spurious response between the two passbands.

**Figure 8:**
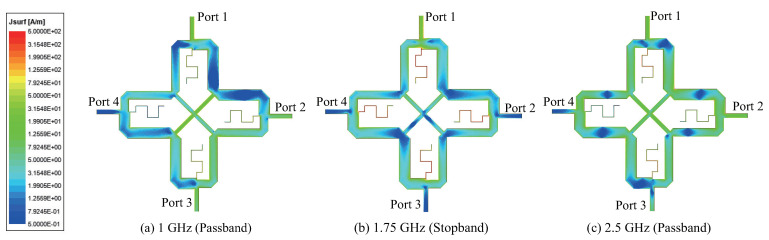
Current distribution of Coupler B ($k_{1}$ = −3 dB and $k_{2}$ = +3 dB).

**Figure 9:**
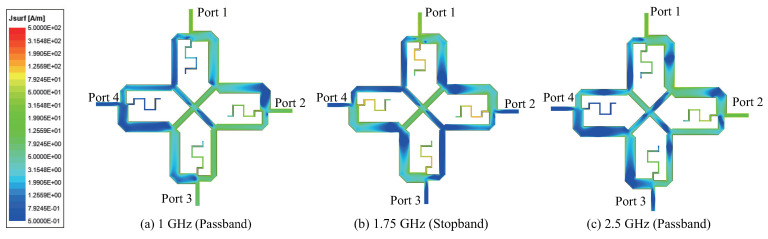
Current distribution of Coupler C ($k_{1}$ = 0 dB and $k_{2}$ = +13 dB).

The surface current distribution for Coupler B is shown in Figure 8 for various frequencies, with Port 1 as input. It shows that at the two passband center frequencies, the current distribution at towards the two output ports (Port 2 and 3) is fairly high and uniform. However, the current distribution towards Port 4 is low, leading to high isolation. At a stopband frequency of 1.75 GHz, the currents are on the stubs, and therefore weak towards the two output ports. The current distribution for Coupler C in Figure 9 is similar to that for Coupler B above, except that at the upper center frequency of 2.5 GHz, current distribution towards Port 3 is relatively weak due to the large power-split ratio of 13 dB.

Table 2 summarizes the measured results and compares our results with those obtained by others. The comparison reveals that the proposed coupler can be designed not only to operate independently in the two bands, but also with the most accurate phase response and the highest directivity. Despite the crossed lines that make it difficult to miniaturize, the couplers still maintain moderate size. Although it is not shown here, the coupler can be designed to have larger than 30 dB of difference in the power-split ratios of two bands, and the measured difference of 13.3 dB in the power-split ratios of the two bands is the largest demonstrated thus far for a dual-band branch-line coupler.

For Coupler A, the measured power-split at the two design frequencies is +3.0 dB and −3.5 dB, which closely corresponds with the ideal and HFSS results. The power-split ratio bandwidths, which are the bandwidths for which the power-split ratios $k_{1}$ and $k_{2}$ are maintained within ±0.5 dB from the designed values, are 8.4% and 2.4%, in which the reflection and isolation remain below −15 dB and −20 dB, respectively. The phase difference between the two outputs was within ±90${}^{\circ }$±1.7${}^{\circ }$ in this band. For Coupler B, the measured power-split ratios are −2.9 dB and +2.8 dB at the two design frequencies. The power-split bandwidths are 7.0% and 1.6% at 1 and 2.5 GHz, respectively, for which the reflection and isolation remain below −15 dB and −20 dB, and the phase difference within ±90${}^{\circ }$±2.2${}^{\circ }$. For Coupler C, the measured power-split ratios are −0.2 dB and +13.1 dB with bandwidths of 6.9% for the lower band and 2.3% for the upper band, in which the reflection and isolation are maintained below −15 dB and −20 dB, respectively. Simultaneously, the phase difference remained within ±90${}^{\circ }$±2.7${}^{\circ }$. All demonstrated couplers show outstanding stopband performance of maintaining all outputs $|S_{21}|$, $|S_{31}|$, and $|S_{41}|$ below −15 dB in a relatively wide to moderate bandwidths of 23.2%, 9.3%, and 7.0% for Coupler A, B, and C, respectively. However, the asymmetric responses of the two bands is an inherent problem of transmission-line based dual-band circuits that provide different properties in the two bands. Further, the asymmetry between $S_{21}$ and $S_{31}$ is a common problem for couplers based on branch-line couplers. Filtering couplers have been reported that is free from this problem, at the cost of independence between the two bands [21,22].

## 4. Discussion

For the bandwidths in which the power-split ratio remained within ±0.5 dB from the designed value, the reflection remained below −15 dB, the directivity above 17 dB, and the phase difference between the two outputs was within ±90${}^{\circ }$±$2.7^{\circ }$ in the worst case for the proposed couplers. Moreover, the open stubs successfully improve the notorious spurious response between the two bands below −15 dB. Most importantly, the coupler is sufficiently versatile to ensure a difference of as large as 13 dB in the power-split ratios of the two bands. The coupler has a relatively small $S_{21}$ and $S_{31}$ bandwidths. This can be improved by cascading a number of couplers, which remains to be investigated.

## 5. Conclusions

Dual-band branch-line couplers with arbitrary power-split ratios based on crossed lines and open-ended stubs are presented. The coupler utilizes crossed lines and open stubs, which allow for a large difference in the power-split as well as frequency ratios. The experimental results for the three dual-band couplers were in excellent agreement with the simulated results, verifying its versatility that a sufficiently wide power-split ratio could be implemented.

## Figures and Tables

**Table 1:** Design parameters of proposed branch-line coupler.

	Characteristic Impedance				Electrical Length @ $f_{1}$			
**Type**	$Z_{1}$	$Z_{2}$	$Z_{3}$	$Z_{4}$	$\theta_{1}$	$\theta_{2}$	$\theta_{3}$	$\theta_{4}$
	**(Ω)**	**(Ω)**	**(Ω)**	**(Ω)**	**(${}^{\circ }$)**	**(${}^{\circ }$)**	**(${}^{\circ }$)**	**(${}^{\circ }$)**
Coupler A	30.6	66.6	31.3	50	52.3	44.7	45.0	51.4
Coupler B	25.1	31.3	52.7	155	59.6	55.3	24.3	51.4
Coupler C	25.6	37.7	41.1	100	63.2	56.7	24.1	51.4

**Table 2:** Summary and comparison of measured results.

Ref.	$f_{}$, $f_{2}$ (GHz)	R. BW † (%)	I. BW ‡ (%)	Directivity (dB)	$k_{}$, $k_{2}$ (dB)	$k_{}$/$k_{2}$ (dB)	$∠\;S_{}-∠\;S_{31}$ (${}^{\circ }$)	Max. $|k_{}/k_{2}|$ * (dB)	Substrate $\epsilon_{}$/*h* (mm)	Size ($\lambda_{0}^{2}$)
[4]	1, 6	>84, >18 ${}^{1}$	>100, >100 ${}^{3}$	21.5, 14.5	0.4, 0.1	0.3	−90.9, −88.0	2.1	3.38/1.524	0.14 × 0.05
	2, 4	>23, >16.8 ${}^{1}$	>23, >11.5 ${}^{3}$	15.8, 13.1	0.5, 2.6	−2.1	−90.7, −93.0			0.64 × 0.11
[5]	1, 2.5	N.A	N.A	N.A	3.3, 2.9	0.4	87.6, −92.7	0.5	2.2/0.79	0.22 × 0.23
	1, 2.5	N.A	N.A	N.A	−2.5, −3.0	0.5	86.2, −98.4			0.23 × 0.21
[6]	2.45, 5.5	10.9, 9.4${\;}^{2,4}$		12.8, 15.8	−0.4, 2.7	−3.1,	88.0, −94.1	3.1	12.88/N.A	0.18 × 0.11
	2.45, 5.5	13.2, 9.2 ${}^{2,4}$		18.4, 16.5	2.8, 2.8	0	87.2, −95.0			0.14 × 0.01
[7]	2.45, 5.2	15.5, 23.5 ${}^{2,4}$	14.1, 15.5 ${}^{2,4}$	20.4, 19.5	3.2, 6.8	−3.6	87.8, 86.9	3.6	2.2/0.508	0.11 × 0.09
[8]	3, 5.5	N.A	N.A	N.A	−0.4, −9.7	9.3	−97.9, −92.4	10.2	2.33/1.57	0.14 × 0.19
	3, 4.5	N.A	N.A	N.A	10.3, 0.1	10.2	−97.3, −90.9			0.16 × 0.16
[9]	1.2, 2.52	24.8, 15.1	21.8, 11.9	>13.2	5.4, 4.8	0.4	88.9, −88.9	0.4	3.55/0.813	0.3 × 0.15
	1.0, 2.0	11.5, 9${\;}^{2,4}$	11, 8.3 ${}^{2,4}$	>15.5	1.5, 1.7	0.2	91.7, −92.8			0.37 × 0.18
[10]	2.4, 5.2	10.8, 28.6 ${}^{2,4}$	75.6, 13.4 ${}^{2,4}$	N.A	9.2, 5.7	3.5	61.9, 79.2	3.5	3.38/1.5	0.32 × 0.1
This work	1, 2.5	14.4, 6.3 ${}^{1,3}$	>65.1, 6.2 ${}^{1,3}$	24.0, 20.6	3.0, −3.5	6.5	90.2, −90.4	13.3	2.2/0.76	0.27 × 0.27
	1, 2.5	27.2, 8.4 ${}^{1,3}$	48.9, 31.1 ${}^{1,3}$	32.1, 17.4	−2.9, 2.8	−5.7	89.6, −91.3			0.28 × 0.3
	1, 2.5	23.5, 7.5 ${}^{1,3}$	26.7, >23.7 ${}^{1,3}$	23.4, 28.6	−0.2, 13.1	−13.3	89.5, −91.6			0.28 × 0.28

†: Reflection BW, ‡: Isolation BW. *: Achievable maximum power-split ratios difference between the two bands. ^1^: (|*S*_11_| < −10 dB), ^2^: (|*S*_11_| < −15 dB), ^3^: (|*S*_41_| < −10 dB), and ^4^: (|*S*_41_| < −15 dB).

## Data Availability

Not applicable.
